# Nutritional status affects inflammatory responses and exacerbates the severity of pulmonary tuberculosis

**DOI:** 10.3389/fcimb.2025.1635870

**Published:** 2025-12-18

**Authors:** Qing Xia, Anbang Wang, Yan Zhang, Jing Meng, Shasha Wu, Panpan Zhu, Zhilong Guo, Jing Hou, Hua Wang, Xueying Liu

**Affiliations:** 1 Third Department of Tuberculosis, Anhui Chest Hospital, Hefei, Anhui, China; 2 Second Department of Oncology, Anhui Chest Hospital, Hefei, Anhui, China; 3 Eight Department of Tuberculosis, Anhui Chest Hospital, Hefei, Anhui, China; 4 General Practice, The Second Affiliated Hospital of Anhui Medical University, Hefei, Anhui, China

**Keywords:** nutritional status, inflammatory response, pulmonary tuberculosis, neutrophil-to-lymphocyte ratio, geriatric nutritional risk index

## Abstract

**Purpose:**

This study aimed to comprehensively assess the impact of nutritional status and inflammatory response on the severity of pulmonary tuberculosis (PTB).

**Methods:**

Hospitalized patients with active PTB were included. Severe PTB was defined as active PTB with ≥3 infected lobes on chest imaging. Nutritional status was determined by the geriatric nutritional risk index (GNRI) and prognostic nutritional index (PNI). Inflammatory markers included monocyte-to-lymphocyte ratio (MLR), neutrophil-to-lymphocyte ratio (NLR), and systemic inflammatory response index (SII). Multivariate logistic regression, receiver operating characteristic (ROC) curves, random forest, and mediation analysis were leveraged to clarify the links of nutritional status and inflammatory response with PTB severity.

**Results:**

337 patients were included. In the fully-adjusted logistic regression model, GNRI (OR: 0.93; 95%CI: 0.90-0.96, P<0.001) and PNI (OR: 0.90; 95%CI; 0.86-0.95, P<0.001) were independent protective factors for severe PTB, whereas NLR (OR: 1.07; 95%CI: 1.01-1.16, P<0.05) and MLR (OR: 3.11; 95%CI: 1.16-9.71, P<0.05) were independent risk factors. No association between SII and severe PTB was found (P>0.05). GNRI mediated 51.64% and 60.58% of the effect of NLR and MLR on PTB, respectively. PNI mediated 70.15% and 76.70% of the effect of NLR and MLR on PTB, respectively. When NLR, MLR, GNRI, and PNI were integrated with traditional clinical indexes, the AUC increased to 0.723 (95% CI: 0.668-0.777).

**Conclusion:**

Nutrition and inflammatory response are significantly associated with PTB severity, and nutritional status mediates the effect of inflammatory response on PTB severity.

## Introduction

Pulmonary tuberculosis (PTB) is a chronic infectious lung disease caused by Mycobacterium tuberculosis (MTB). As the Global Tuberculosis Report 2024 stated (Global tuberculosis report 2024, 2024), there were 10.8 million new cases of PTB globally in 2023, with an incidence of 134/100,000. The 30 high PTB-burden countries accounted for 87% of the total global cases, and China ranked third in terms of estimated PTB incidence, accounting for 6.8% of the global incidence. In 2023, new PTB patients in China reached 741,000, slightly lower than the previous year. The estimated number of multidrug-resistant/rifampicin-resistant PTB patients was 29,000, ranking fourth worldwide. About 1%-3% of PTB patients may progress to severe PTB. These patients have extensive lung lesions and obvious symptoms of tuberculosis poisoning. Severe cases may experience respiratory and circulatory failure and even life-threatening conditions and have a higher mortality rate than most patients with common PTB. As a key source of infection, their high volume and long duration of bacteria excretion, if not controlled effectively and timely, can result in the widespread spread of MTB and an increase in the infection risk in the general population. Improving the outcomes of critically ill PTB patients is crucial to reducing PTB-related mortality.

Inflammatory response and nutritional status are crucial for clinical outcomes of PTB patients. The acute inflammatory response triggered by MTB infection helps to clear the pathogen, but excessive or persistent chronic inflammation may lead to tissue damage. A persistent inflammatory state may accelerate the activation and spread of MTB, leading to more severe lesions, such as cavity formation and pleurisy, thus complicating the treatment. Chronic inflammation may disrupt drug metabolism and alter drug distribution, thereby reducing the efficacy of anti-tuberculosis drugs. While certain anti-inflammatory treatments (e.g., glucocorticoids) may alleviate symptoms, they may also mask changes in the disease, which is not conducive to an accurate assessment of the response to treatment, and can cause immunosuppression, thus increasing the risk of PTB infection (Pan et al., 2022). Adequate nutrition is essential for maintaining normal immune function. Micronutrients are essential in immune cell production, maintenance of activity, and signal transduction. Favorable nutritional status helps to ensure optimal absorption and metabolism of anti-tuberculosis drugs, thereby increasing the treatment efficacy. Conversely, malnutrition may lead to reduced drug bioavailability and weakened immune responses, making the body more susceptible to MTB infection, slowing recovery, and even triggering adverse reactions. While existing studies have explored the links among nutritional status, inflammatory response, and PTB severity, cohort studies that have simultaneously explored the combined effects of nutrition and inflammatory response on TB severity are still limited. There is still a paucity of comprehensive investigations into how nutrition and inflammation affect PTB severity.

Therefore, this study aimed to clarify the links of nutrition and inflammatory response with PTB severity (single and combined effects), as well as to determine whether the nutritional status could mediate the link between inflammatory response and PTB severity.

## Materials and methods

### Cohorts

This study retrospectively included 337 hospitalized patients with active PTB from January 2023 to January 2025 in Anhui Chest Hospital. Patients meeting the diagnostic criteria of active PTB (WS288-2017) were included. Exclusion criteria covered: 1) patients with malignant tumors; 2) combined rheumatic immune diseases; 3) pregnant patients. The study was approved by the Medical Ethics Committee of Anhui Chest Hospital (KJ2025-002).

### Data collection

Baseline information, personal history, underlying diseases, laboratory indices, and lung imaging of each patient were collected. Baseline information included sex, age, and body mass index (BMI). Personal history included smoking history and alcohol consumption. Underlying diseases encompassed diabetes mellitus (DM), hypertension, and chronic obstructive pulmonary disease (COPD). Routine blood markers included lymphocyte count (Lym), monocyte count (Mon), neutrophil count (Neu), platelet count (Pla), and ultrasensitive C-reactive protein (Ultra-CRP). Biochemical parameters included serum albumin (SA), triglycerides (TG), total cholesterol (TC), prealbumin, globulin, blood urea nitrogen (BUN), and creatinine (CRE); coagulation function included D-dimer and fibrinogen. Imaging characteristics of the lungs referred to the number of lobes infected with PTB foci and the complicated presence of PTB from other sites. About 3 mL of fasting venous blood was drawn from superficial veins, such as the elbow, with a disposable vacuum blood collection needle and injected into an anticoagulant-containing vacuum tube, which was mixed thoroughly and sent for routine blood tests.

### PTB definition

Lymphopenia and monocytosis may trigger an inflammatory storm in patients with severe PTB (Wang et al., 2023). Lymphopenia impedes the immune response and leads to systemic immunosuppression, which is linked with PTB mortality (Li et al., 2023). PTB severity is associated with anemia and inflammatory immune profiles (Ashenafi et al., 2023). Research indicates that the number of lung lobes affected serves as a surrogate indicator of severity (Wang et al., 2025). Another study suggested that the infected lung lobes ≥3 was the sole independent indicator of shorter survival in PTB populations (Lu et al., 2024). Hence, in this study, severe PTB was defined as active PTB with infected lobes ≥3 on chest imaging. All included participants were categorized into a severe PTB group (infected lobes ≥3) (n=198) and a non-severe PTB group (infected lobes <3) (n=139) based on imaging tests.

### Assessment of nutritional status and inflammatory response

Two common malnutrition assessment tools, geriatric nutritional risk index (GNRI) and prognostic nutritional index (PNI), were utilized to evaluate the nutritional status.

GNRI: [1.489 × SA (g/L)] + 41.7 × (current weight/ideal body weight); ideal body weight = height (cm) - 100 – [height (cm) - 150)/(4 (male), 2.5 (female)].

PNI: SA (g/L) + 5 × peripheral blood Lym (× 10^9^/L).

For inflammatory response, monocyte-to-lymphocyte ratio (MLR), neutrophil-to-lymphocyte ratio (NLR), and systemic inflammatory response index (SII) were calculated. SII = Pla (×10^9^/L) × Neu (×10^9^/L)/Lym (×10^9^/L) (Xie et al., 2022).

### Treatment of missing values

To minimize bias due to sample exclusion, the percentage of missing values was estimated. For variables with a percentage of missing values < 10%, the missing values were predicted using a multiple interpolation method based on the random forests model, and the average of the five outcomes was used as the final result. Variables with a percentage of missing values > 10% were eliminated.

### Statistical analysis

Statistical analyses were performed in R software 4.4.2 unless otherwise noted. Continuous variables not in normal distribution were depicted as median (M) and upper and lower quartiles (P25, P75) and were compared utilizing nonparametric tests. Categorical variables were depicted as the number and percentage (%) and compared with the chi-square test. To prevent multicollinearity, variables with a variance inflation factor > 4 were excluded from the model. Logistic regression models were leveraged to estimate the odds ratios (ORs) and 95% confidence intervals (CIs) to assess the links of nutritional status and inflammatory response with PTB severity. First, univariate analyses were performed, and sex, age, DM, hypertension, smoking history, and alcohol consumption history were adjusted in Model 2; TG, TC, globulin, D-dimer, BUN, CRE, fibrinogen, consolidated bronchial tuberculosis, and consolidated extrapulmonary tuberculosis were further adjusted in Model 3 based on Model 2. The area under the receiver operating characteristic (ROC) curve (AUC) was calculated to appraise the predictive ability of individual and combined metrics of nutritional or inflammatory response. P-value <0.05 (two-sided) was considered statistically significant.

### Random forests model

Random forest models were constructed using the default settings of the randomForest V4.7-1.1 package with the indicators related to nutritional or immune status, individually or in combination, to explore the indicators important for classifying severe PTB. Parameter importance was assessed using SHapley Additive exPlanations with the DALEX V2.4.3 package and displayed using the shapviz V0.9.0 package. Patients were randomized into the training and validation sets in a ratio of 7:3.

### Mediation analysis

To assess whether nutritional status mediated the effect of the inflammatory response on PTB severity or PTB-infected lung lobes, the mediate function in the R package was utilized to conduct causal mediation analyses. The total, direct, and indirect effects of nutritional status on the link between inflammatory response on TB severity were estimated. The results were validated by 1000 simulated bootstrap repetitions.

## Results

### Clinical features

**Table 1** shows the clinical features. 337 individuals (median age: 56 years) were included, with 237 (70%) males and 100 (30%) females. 198 individuals had severe PTB, accounting for 58.75% of all participants. Severe PTB individuals were more likely to be older men with DM than general PTB individuals, regardless of whether they had previously smoked or consumed alcohol, or whether they had underlying medical conditions, such as hypertension and COPD. Severe PTB individuals exhibited lower BMI, SA, prealbumin, GNRI, and PNI; lower lymphocytes, higher neutrophils, monocytes, ultra-CRP, D-dimer, and fibrinogen, higher MLR and PLR, and lower SII (P<0.05). These results suggest that severe PTB patients have poorer nutritional status and more severe inflammatory responses than those with PTB.

**Table 1:** Characteristics of participants by PTB severity (n=337).

1	Variables	Total (n = 337)	PTB(n = 139)	SPTB(n = 198)	p
2	gender, n (%)				0.046
3	male	237 (70)	89 (64)	148 (75)	
4	female	100 (30)	50 (36)	50 (25)	
5	age (year)	56 (36, 72)	50 (30.5, 62)	61 (47, 73)	< 0.001
6	diabetes, n (%)				0.045
7	no	246 (73)	110 (79)	136 (69)	
8	yes	91 (27)	29 (21)	62 (31)	
9	hypertension, n (%)				0.631
10	no	271 (80)	114 (82)	157 (79)	
11	yes	66 (20)	25 (18)	41 (21)	
12	copd, n (%)				0.198
13	no	317 (94)	134 (96)	183 (92)	
14	yes	20 (6)	5 (4)	15 (8)	
15	smoking_history, n (%)				0.225
16	no	262 (78)	103 (74)	159 (80)	
17	yes	75 (22)	36 (26)	39 (20)	
18	drinking_history, n (%)				1
19	no	311 (92)	128 (92)	183 (92)	
20	yes	26 (8)	11 (8)	15 (8)	
21	height (cm)	167 (160, 171)	168 (161.5, 172.5)	165 (160, 170)	0.154
22	weight (kg)	55 (48, 64)	58.5 (51.75, 65.25)	52 (45, 61.75)	< 0.001
23	bmi	20.07 (18.06, 22.1)	21.26 (19.13, 23.51)	19.5 (17.48, 21.74)	< 0.001
24	serum_albumin (g/L)	36.6 ± 6.3	39.08 ± 5.92	34.86 ± 5.98	< 0.001
25	lymphocyte_count (*109/L)	1.19 (0.85, 1.62)	1.38 (1.04, 1.75)	1.08 (0.74, 1.49)	< 0.001
26	triglyceride (mmol/L)	1.05 (0.78, 1.36)	1.05 (0.74, 1.45)	1.05 (0.8, 1.33)	0.802
27	total_cholesterol (mmol/L)	4.12 (3.45, 4.76)	4.21 (3.54, 5.03)	4.06 (3.42, 4.66)	0.067
28	prealbumin (mg/L)	171.98 ± 71.02	199.91 ± 73.61	152.37 ± 62.21	< 0.001
29	globin (g/L)	30.3 (27, 34.1)	29.1 (26.4, 32.8)	31.2 (27.75, 34.88)	< 0.001
30	neutrophil_count (*109/L)	3.95 (2.94, 5.06)	3.65 (2.76, 4.43)	4.19 (3.11, 6.01)	< 0.001
31	monocyte_count (*109/L)	0.43 (0.33, 0.58)	0.4 (0.32, 0.51)	0.44 (0.33, 0.61)	0.016
32	blood_platelet_count (*109/L)	237 (186, 304)	229 (190.5, 276.5)	244 (182.25, 318.75)	0.198
33	hypersensitive_c_reactive_protein (mg/L)	10 (2.74, 36.3)	4.76 (1.06, 17.07)	14.81 (6.33, 51.7)	< 0.001
34	d_dimer (mg/L)	0.46 (0.29, 0.95)	0.37 (0.15, 0.6)	0.58 (0.34, 1.39)	< 0.001
35	blood_urea_nitrogen (umol/L)	5.3 (4, 6.7)	5.2 (3.85, 6.6)	5.35 (4.03, 6.9)	0.464
36	creatinine (umol/L)	60.5 (51.4, 72.5)	61.3 (51.5, 73.2)	60.45 (51.47, 71.4)	0.816
37	fibrinogen (g/L)	3.85 (2.87, 4.72)	3.4 (2.62, 4.56)	4.12 (3.15, 4.97)	< 0.001
38	cavity, n (%)				0.007
39	no	210 (62)	99 (71)	111 (56)	
40	yes	127 (38)	40 (29)	87 (44)	
41	combined_bronchial_tuberculosis, n (%)				0.202
42	no	283 (84)	112 (81)	171 (86)	
43	yes	54 (16)	27 (19)	27 (14)	
44	with_extrapulmonary_tuberculosis, n (%)				0.503
45	no	292 (87)	123 (88)	169 (85)	
46	yes	45 (13)	16 (12)	29 (15)	
47	ideal_weight, Median (Q1,Q3)	61.25 (57.2, 65.75)	62 (57.35, 66.65)	61.25 (57.2, 65)	0.323
48	GNRI, Mean ± SD	91.31 ± 11.7	96.31 ± 10.42	87.79 ± 11.28	< 0.001
49	PNI, Mean ± SD	42.87 ± 7.92	46.19 ± 7.43	40.53 ± 7.42	< 0.001
50	SII, Median (Q1,Q3)	783.62 (440.79, 1512.61)	594.07 (362.69, 960.85)	1050.12 (553.01, 1822.01)	< 0.001
51	NLR, Median (Q1,Q3)	3.38 (2.15, 5.24)	2.73 (1.72, 3.97)	4.42 (2.64, 6.93)	< 0.001
52	MLR, Median (Q1,Q3)	0.38 (0.25, 0.58)	0.29 (0.22, 0.44)	0.46 (0.29, 0.68)	< 0.001

### Links of nutritional status and inflammatory response with PTB severity

**Table 2** demonstrates logistic regression analysis on the link between nutritional/inflammatory indicators and severe PTB. GNRI [Model 1: OR(95%CI) 0.93(0.91-0.95); Model 2: 0.93(0.90-0.95); Model 3: 0.93(0.90-0.96); all P<0.001] and PNI [Model 1: 0.90(0.87-0.93); Model 2: 0.91(0.87-0.94); Model 3: 0.90(0.86-0.95); all P<0.001] were greatly positively correlated with severe PTB risk. NLR (Model 1: 1.12(1.05-1.22), P = 0.002; Model 2: 1.09(1.02-1.18), P = 0.017; Model 3: 1.07(1.01-1.16), P = 0.045) and MLR (Model 1: 6.10 (2.59-16.1), P<0.001; Model 2: 4.53(1.85-12.5), P = 0.002; Model 3: 3.11(1.16-9.71), P = 0.036) was negatively correlated with severe PTB risk. No association was found between SII and severe PTB (P>0.05).

**Table 2:** Association between nutritional/inflammatory indicators and severe PTB.

Variables	Model 1		Model 2		Model 3	
	OR(95%CI)	P	OR(95%CI)	P	OR(95%CI)	P
Nutritional status						
GNRI	0.93(0.91-0.95)	<0.001	0.93(0.90-0.95)	<0.001	0.93(0.90-0.96)	<0.001
PNI	0.90(0.87-0.93)	<0.001	0.91(0.87-0.94)	<0.001	0.90(0.86-0.95)	<0.001
Inflammatory condition						
SII	1.00(1.00-1.00)	0.002	1.00(1.00-1.00)	0.006	1.00(1.00-1.00)	0.055
NLR	1.12(1.05-1.22)	0.002	1.09(1.02-1.18)	0.017	1.07(1.01-1.16)	0.045
MLR	6.10(2.59-16.1)	<0.001	4.53(1.85-12.5)	0.002	3.11(1.16-9.71)	0.036
PLR	1.00(1.00-1.01)	<0.001	1.00(1.00-1.00)	<0.001	1.00(1.00-1.00)	0.009

Model 1, unadjusted.

Model 2, gender, age, diabetes mellitus, hypertension, smoking history, alcohol consumption history.

Model 3, in addition to Model2, further adjusted for triglycerides, total cholesterol, globulin, D-dimer, blood urea nitrogen, creatinine, fibrinogen, combined bronchial tuberculosis, combined extrapulmonary tuberculosis.

### ROC analyses

**Figure 1:**
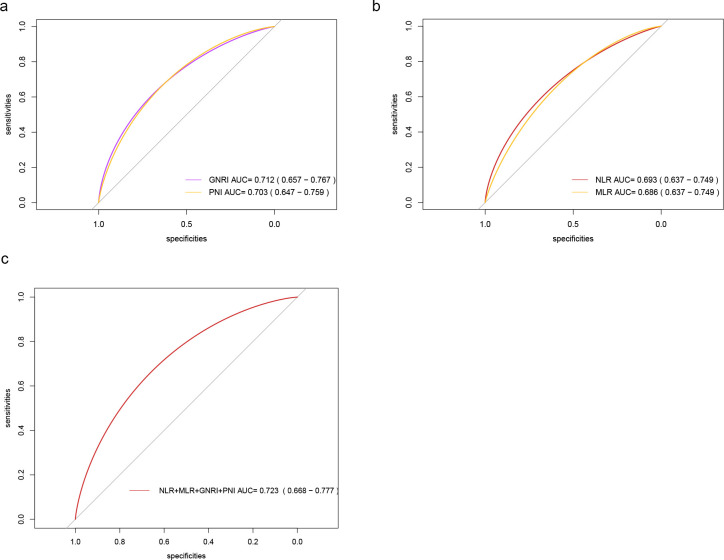
ROC curves of each index for severe PTB diagnosis. **(a)** ROC curves of GNRI and PNI. **(b)** ROC curves of NLR and MLR. **(c)** ROC curves of NLR+MLR+GNRI+PNI.

Among the single indicators, GNRI had the best predictive efficacy (AUC = 0.712, 95% CI: 0.657-0.767), followed by PNI (AUC = 0.703, 95% CI: 0.647-0.759). The AUC of NLR and MLR was 0.693 (0.637-0.749) and 0.686 (0.637-0.749), respectively; the AUC of the combined model (NLR+MLR+GNRI+PNI) was elevated to 0.723 (95%CI: 0.668-0.777), greatly higher than the conventional clinical index (**Figure 1**). The AUC value of both models was higher than that of the model based only on common clinical indexes, suggesting that these nutritional and inflammatory indicators have better abilities to distinguish severe PTB patients from common PTB individuals than common clinical indexes.

### Random forest models

**Figure 2:**
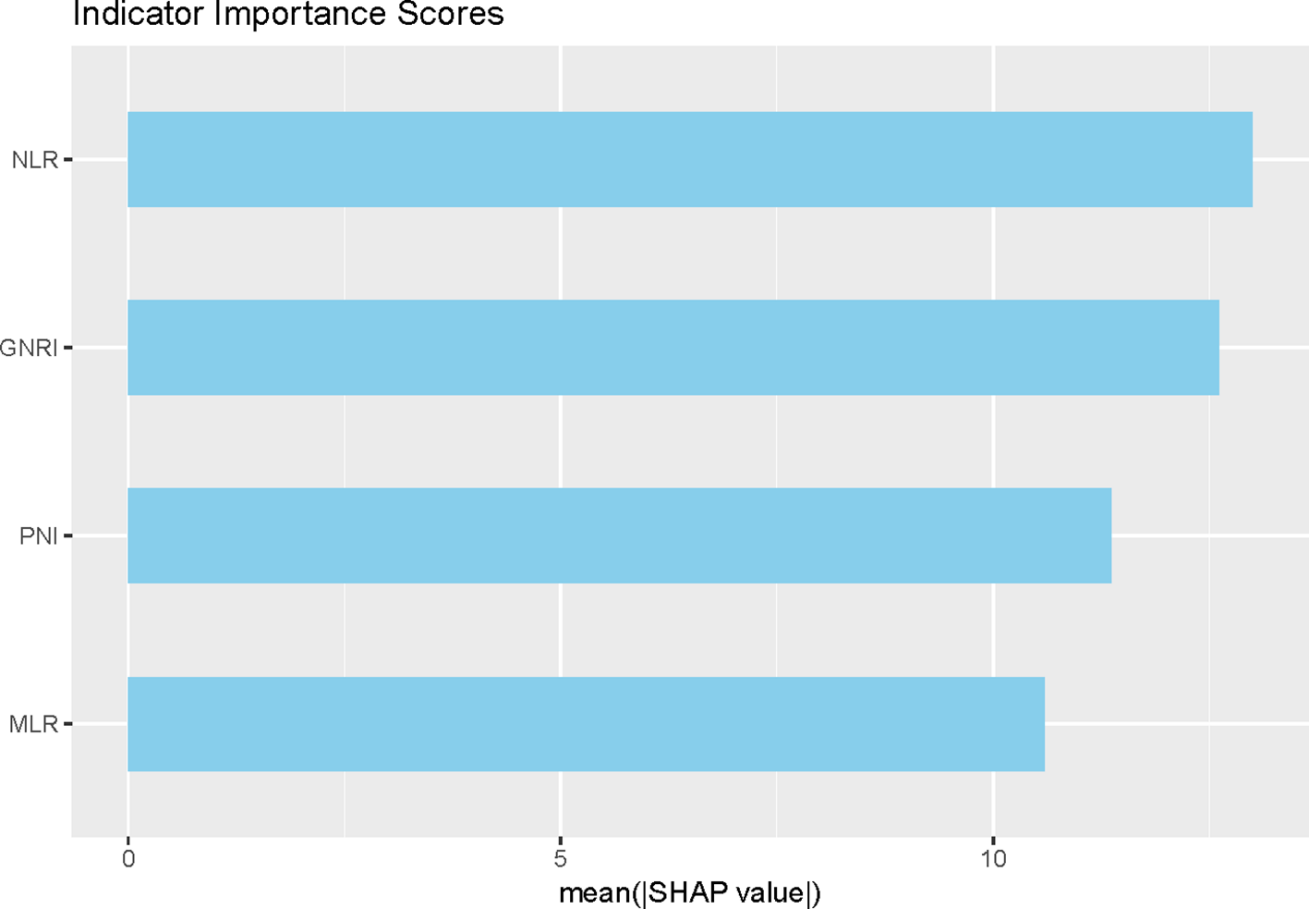
Ranking the importance of nutritional and inflammatory indicators in the diagnosis of PTB severity.

In addition, we found that a machine learning algorithm combining nutritional and inflammatory metrics could discriminate between patients with severe PTB and those without severe PTB. Incorporating nutritional and inflammatory indicators into the model, the ranking of parameters contributing most to the random forest was NLR>GNRI>PNI>MLR. Thus, NLR and GNRI were considered important indicators of nutritional status and inflammatory response related to PTB severity (**Figure 2**).

### Mediation analysis

**Figure 3:**
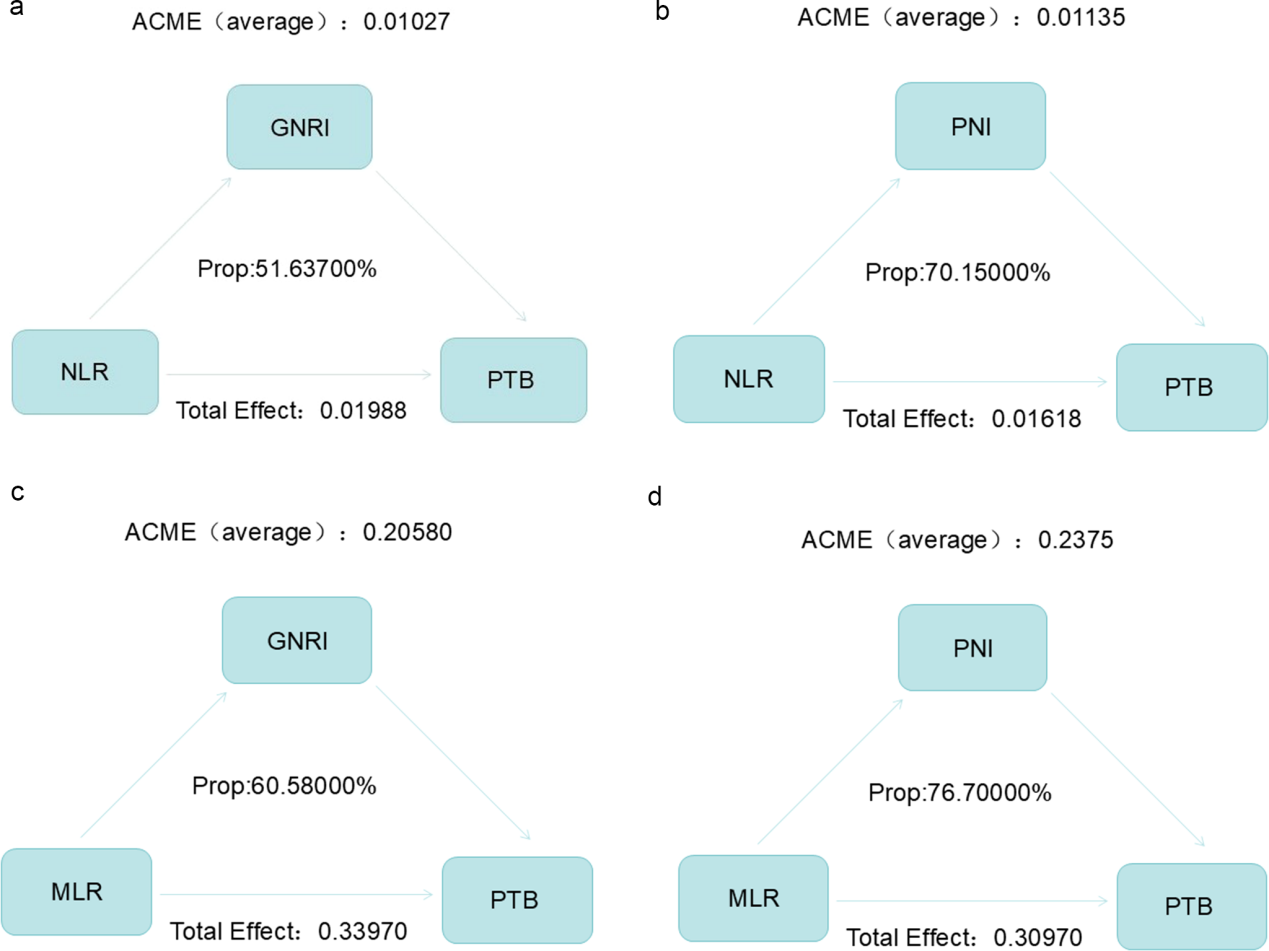
The inflammatory response may influence PTB severity by modulating the nutritional status. **(a)** Mediation effect of GNRI on the link between NLR and PTB severity. **(b)** Mediation effect of PNI on the link between NLR and PTB severity. **(c)** Mediation effect of GNRI on the link between MLR and PTB severity. **(d)** Mediation effect of PNI on the link between MLR and PTB severity. P indicates the mediation effects of each metabolite.

**Figure 3** shows how nutrition and inflammation work together to influence PTB severity. Mediation analysis revealed that GNRI mediated 51.637% of the link between NLR and PTB, and PNI mediated 70.15% of the link between NLR and PTB. GNRI mediated 60.58% of the link between MLR and PTB, and PNI mediated 76.70% of the link between MLR and PTB. Mediation analysis suggested that the inflammatory response may influence PTB severity by modulating the nutritional status.

## Discussion

This study examined the links of nutrition and inflammatory response with PTB severity and explored how nutritional status mediated the inflammatory response and exacerbated PTB severity. The findings illustrated that nutritional and inflammatory responses were considerably associated with PTB severity. GNRI and PNI were protective factors for PTB severity, while NLR and MLR were risk factors. Additionally, nutritional factors mediated the effect of inflammatory factors on PTB severity.

Nutritional status is recognized as a principal factor for the prognosis of multiple diseases (Huang et al., 2024). In inflammatory bowel disease, approximately 75% of patients with active lesions are malnourished (Jabłońska, 2025). The severity of malnutrition is a key risk factor for disease progression and overall low survival in patients with pancreatic cancer (Pires et al., 2024). Malnutrition is also closely linked with PTB development. Malnourished patients have a reduced ratio of helper T-cells/suppressor T-cells and impaired cellular immunity, leading to susceptibility to PTB. Disturbed appetite mediator ratios in PTB patients lead to poor appetite and malnutrition. Numerous studies have demonstrated that nutritional status or immune function is closely associated with the prognosis of PTB. SA levels, TC content, and lymphocyte counts can serve as indicators for assessing the prognosis of PTB (Tan et al., 2024). Immune function mediates the impact of nutritional status on the severity of tuberculosis (Lu et al., 2024). However, there remains a lack of studies that simultaneously integrate indicators of nutritional status and inflammatory response to systematically assess their impact on the severity of tuberculosis, particularly research employing mediation analysis to quantify and elucidate the potential underlying mechanisms between the two. GNRI (Huang et al., 2025) is a simple and easy clinical tool. It integrates weight, height, and SA levels and is a validated tool with prognostic significance in diverse patient populations, including patients with renal disease (Zhao et al., 2024; Zou et al., 2024), pancreatic cancer (Grinstead and Yoon, 2025), and COPD (Zhang et al., 2024). It can be used to assess nutritional risk in old patients (Haas et al., 2023) and is a potential prognostic marker in diverse settings, including malignancies.

PNI is a simple and effective indicator for assessing nutritional status and integrates Lym and albumin. Lymphocytes mainly mediate adaptive immunity, with regulatory or protective roles. Low lymphocyte count usually infers an unfavorable immune status, while SA levels usually reflect nutritional status. An optimal immune response requires adequate nutrition, and poor nutritional status is linked with inflammation and oxidative stress, which may disrupt the immune system (Iddir et al., 2020; Xu et al., 2024). Low PNI predicts low survival in cancer patients and correlates with TNM staging (Ma et al., 2023; Liu et al., 2024). PNI has been extensively studied in chronic diseases, like vascular and renal diseases, with notable impacts on disease progression and poor outcomes (Kaller et al., 2022). PNI and GNRI can predict poor outcomes after spinal surgery (Huang et al., 2024). Under the subgroups of nutritional levels defined by BMI or GNRI, malnourished PTB patients have a higher risk of all-cause mortality than those who are not malnourished (Xu et al., 2022). GNRI appears to better predict all-cause mortality risk than BMI, yet without statistical significance. Altogether, these findings support that GNRI and PNI may be key nutritional indicators for PTB severity.

Both inflammation and nutritional status are critical in forecasting all-cause mortality in adults (Jia et al., 2024). Several studies have identified NLR as a predictor for various systemic inflammatory diseases. For example, elevated NLR is a risk factor for acute exacerbation of COPD (Cai et al., 2024). High levels of baseline NLR are linked with poor prognoses for immunotherapy in individuals with advanced hepatocellular carcinoma (Du and Huang, 2024). Neutrophils are the first responders to infection and are crucial in eliminating pathogens. However, excessive activation of neutrophils may damage tissue and aggravate PTB. Lymphocytes are responsible for regulating the immune response and maintaining immune homeostasis, and a decrease in lymphocyte count or function may weaken the immune response and accelerate PTB progression, leading to unfavorable outcomes.

There are complex bidirectional regulatory mechanisms between nutritional status and inflammatory response. Malnutrition weakens the immune system, making it difficult for the immune system to effectively defend itself against pathogens, thereby triggering or exacerbating inflammatory responses. For example, short of SA disrupts the production, differentiation, and function of immune cells and weakens the ability to clear MTB, leading to persistent and worsening inflammation, ultimately increasing PTB severity. Conversely, an excessive or prolonged inflammatory response will consume substantial amounts of nutrients, which further worsens the patient’s nutritional status and creates a vicious cycle. This study confirms the mediating role of nutritional status, which suggests that the key to breaking this vicious cycle lies in improving nutritional status. Improving the nutritional status of patients has significant application as an effective strategy to optimize the therapeutic outcome of PTB. Traditional PTB treatment mainly focuses on anti-tuberculosis drugs, but many patients suffer from insufficient nutritional intake and absorption disorders due to long-term illness and side effects, which affect treatment adherence and efficacy. In addition, a favorable nutritional status can help reduce the adverse reactions caused by anti-tuberculosis drugs, such as gastrointestinal discomfort and liver injury, refine the quality of life, facilitate better cooperation with the treatment, and further enhance the therapeutic effect. Improving patients’ nutritional status can effectively reduce the severity and infectiousness of PTB, decrease the number of infectious sources, and help control its spread.

Our study provides evidence that nutritional status and inflammatory response are significantly associated with PTB severity and that nutritional factors mediate the effect of inflammatory factors on PTB severity. Improving the nutritional level of patients may decrease their inflammatory response and reduce the risk of severe PTB. However, this study has certain limitations. The specific mechanism of action of different nutrients during PTB has not been thoroughly investigated. Further detailed investigation is warranted to clarify the effects of various nutrients on inflammatory response and the therapeutic efficacy of PTB. Second, the sample size is limited, and the findings need validation in a larger cohort. In addition, this study identified a history of smoking and alcohol consumption in patients. However, part of the history was not recorded by clinicians, which may have affected our identification. Moreover, this study is cross-sectional in nature and cannot directly infer causal relationships. Future prospective cohort studies will be designed to follow up and clarify the causal pathways among these variables. The study population primarily consisted of hospitalized tuberculosis patients, which may have overestimated the strength of the association between malnutrition, inflammatory responses, and disease severity. Future research should validate these findings in broader populations to enhance their clinical applicability. Furthermore, the number of affected lung lobes is a commonly used radiographic indicator. However, there is a lack of standardized scoring systems for assessing the severity of PTB. The definition provided in this paper focuses solely on radiographic extent. Future efforts will be dedicated to developing and validating multidimensional scoring systems for evaluating the severity of PTB.

## Conclusion

Overall, our study provides evidence that nutritional status and inflammatory response are significantly associated with PTB severity and that nutritional factors mediate the effect of inflammatory factors on PTB severity. These results highlight the importance of focusing on nutritional status and inflammatory response while diagnosing and treating PTB patients. Proactive nutritional supplementation helps to enhance the prognosis of PTB patients.

## Data Availability

The data of this study were derived from inpatients with active pulmonary tuberculosis admitted to Anhui Chest Hospital between January 2023 and January 2025. The study was approved by the hospital's Ethics Committee (approval number: KJ2025-002) and informed consent was obtained from all patients. The original data have not been made public due to patient privacy protection. Qualified researchers can obtain the data by submitting an application to the scientific research management department of the hospital.
